# First report on nasal myiasis in an alpaca “*Vicugna pacos*” – a case report

**DOI:** 10.1186/s12917-018-1706-7

**Published:** 2018-12-04

**Authors:** Teresa Maria Punsmann, Lucie Marie Grimm, Carolin Reckmann, Cornelia Schwennen, Matthias Gerhard Wagener, Martin Ganter

**Affiliations:** Clinic for Swine, Small Ruminants and Forensic Medicine, University of Veterinary Medicine Hannover, Foundation, Hannover, Germany

**Keywords:** Alpaca, New world camelids, Bot flies, Treatment, Doramectin, Germany

## Abstract

**Background:**

An infestation of bot fly larvae causes myiasis which is known to cause respiratory symptoms in ruminants. There are reports of bot fly larvae in llamas, but to our knowledge there are no previous reports of nasal myiasis due to bot flies in alpacas (“*Vicugna pacos*”).

**Case presentation:**

The following case report describes a neutered male alpaca showing sneezing and mild nasal discharge. Endoscopic examination of the upper respiratory tract revealed bot fly larvae in one nostril. After treatment with doramectin, there was no evidence of living bot fly larvae visible in the nostril.

**Conclusion:**

Bot fly larvae should be considered as a potential cause of respiratory symptoms in alpacas. In the present case, a treatment with doramectin was successful.

## Background

In New World camelids bot flies play a role causing parasite-induced respiratory symptoms [1]. However, case reports of myiasis due to bot flies in New World camelids are rare. All reports published to date deal with nasal bots in llamas. There are, in total, four described cases of bot fly larvae in llamas in the United States and South America [2–4]. The animals were presented because of respiratory symptoms like sneezing and nasal discharge, which were not responsive to antibiotic treatment. In cases where the causative species could be isolated, *Oestrus ovis* (sheep and goat bot fly) [3] and *Cephenemyia* spp. (deer bot flies) were identified [4]. In two cases, the species remained unknown [2].

*Oestrus ovis* is a common parasite in sheep and goats. The adult fly shoots larvae onto the area around the nostrils of the affected animals, whence the larvae move into the nasal cavity where they become mature. When they become the third instar larvae they fall to the ground and pupate in the environment [1]. *Cephenemyia* spp. larvae are shot by the flies directly into the nasal cavity where they remain until they reach the third larval stage. The third instar larvae is sneezed out into the environment where they pupate [1].

*Cephenemyia* spp*.* lead to worse reactions in New World camelids than in deer. In the nasal meatus a granulomatous swelling evolves [1, 4].

Knowledge about the life cycle of bot flies in llamas and alpacas is insufficient and further investigations are needed.

In Old World camelids myiasis can be caused by *Cephalopina titillator* [5]. Infections of New World camelids with this species have not been described.

As there are only few described cases of myiasis caused by bot flies in New World camelids recommendations for treatment are rare. In one llama [3] bot fly larvae were identified during necropsy after the animal had died due to haemorrhagic pneumonia. Therefore, no specific treatment was provided. The other affected animals were treated with ivermectin 0.2 mg/kg. In one llama the treatment had to be repeated and combined with local ivermectin administration in order to kill the larvae [2]. One llama, which had been successfully treated subcutaneously with ivermectin, died shortly afterwards due to unknown reasons [2]. To date a treatment with doramectin against bot fly larvae in New World camelids has not been described, while in sheep it is described as an effective drug against *Oestrus ovis* at a dosage of 200 μg/kg [6].

This case report gives the first clinical description of bot flies in an alpaca.

### Case description

A five-year-old neutered male huacaya-alpaca showing sneezing for three weeks was presented to the Clinic for Swine, Small Ruminants and Forensic Medicine, University of Veterinary Medicine Hannover, Foundation, Germany. The alpaca was privately owned and kept on pasture together with four female alpacas. The sneezing was noticed for the first time by the owner about two to three weeks before presenting the animal to the clinic. He reported sneezing fits lasting up to two hours. Apart from that, the general condition of the animal was good. The four female alpacas did not show any symptoms to the author’s knowledge.

**Fig. 1:**
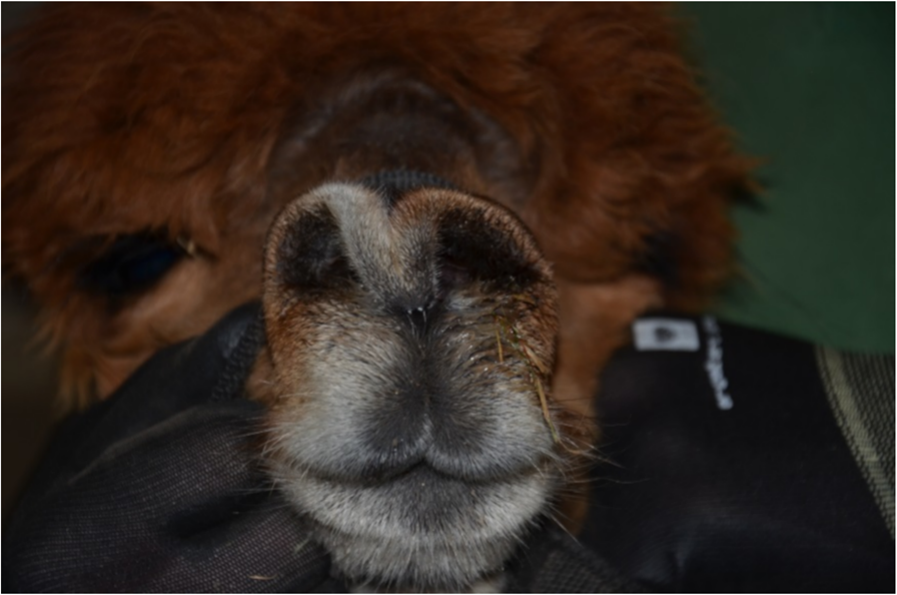
The affected alpaca showing serous nasal discharge and impeded breathing

The alpaca had serous, clear nasal discharge coming out of both nostrils. During examination sneezing could be triggered by applying pressure to the bridge of the nose. The distending of the nostrils indicated that breathing was impeded (Fig. 1). Auscultation of the lung revealed physiologically mild respiratory sounds on both sides.

The analysis of blood samples and faeces showed mild anaemia, granulocytosis and lymphopenia (Table 1). Eosinophils were not increased above the upper reference limits [7]. Clinical chemistry revealed slight hyperproteinaemia, hyperalbuminaemia, hypercalcaemia and hypophosphataemia (Table 1). In the faecal sample a very low number of gastrointestinal nematode eggs was found.

**Table 1:** Haematology and clinical chemistry of the alpaca

	Unit	Reference	Patient
Haematology			
Haemoglobin	g/L	127–166	124
PCV	L/L	0.29–0.37	0.28
MCHC	g/L	414–459	443
Leucozytes	G/L	9.8–15.8	14.9
Neutrophils	G/L	4.5–9.3	10.9
Band neutrophils	G/L	0–0.1	0.07
Lymphocytes	G/L	1.7–4.5	1.1
Monocytes	G/L	0.1–0.7	0.4
Eosinophils	G/L	1–3.6	2.2
Basophils	G/L	0–0.3	0.2
Neutrophils	%	44–70.5	73
Band neutrophils	%	0–1	0.5
Lymphocytes	%	15.2–31	7.5
Monocytes	%	2–5.5	2.5
Eosinophils	%	8–27	15
Basophils	%	0–2	1.5
Clinical chemistry			
Total protein	g/L	59.9–69.1	74.3
Albumin	g/L	31.3–38.6	46.7
Creatinine	μmol/L	97–167	142
Urea	mmol/L	5.2–9.7	4.9
Calcium	mmol/L	2.2–2.5	2.7
Phosphorus	mmol/L	1.6–3	1.5

References from [7]. Only small deviations from reference range were found in the alpaca affected by bot fly larvae

**Fig. 2:**
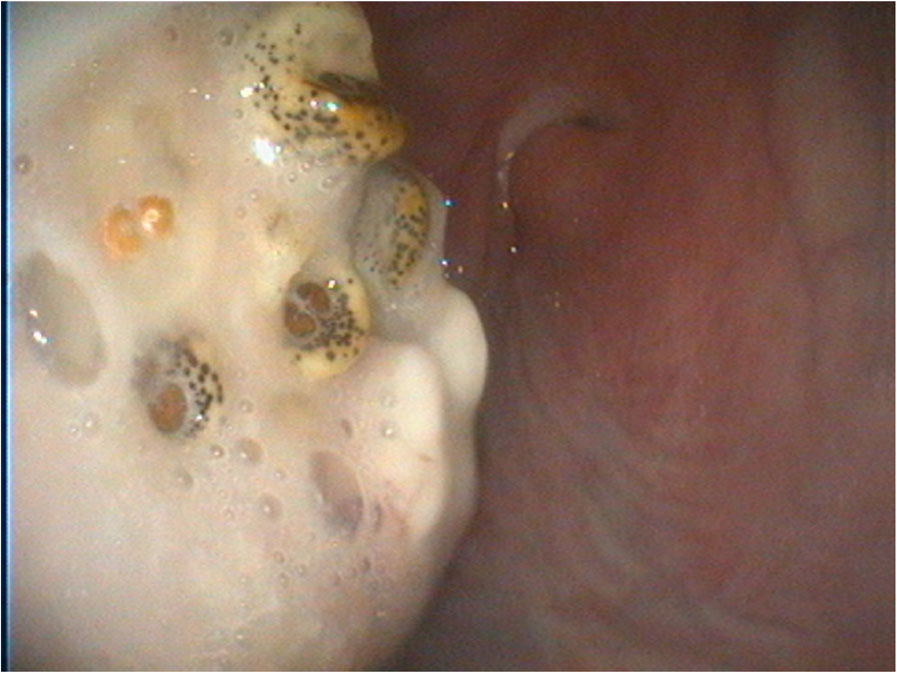
Endoscopic picture of the right ventral meatus from the affected alpaca before treatment. A soft tissue mass with moving larvae was found

Endoscopic examination of the nose was carried out. Due to the tension of the alpaca, the examination was conducted under general anaesthesia (0.4 mg/kg xylazine [Xylavet 20 mg/ml®, CP-Pharma, Burgdorf, Germany], 4 mg/kg ketamine [Ketamidor 100 mg/ml®, WDT, Garbsen, Germany]) [1], [8] and local anaesthesia of the nostrils (Procainhydrochlorid, Epinephrin [Isocain ad us. vet.®, Selectavet, Dr. Otto Fischer GmbH, Weyarn/Holzolling, Germany]). The endoscope was inserted approximately 15 cm into the right ventral nasal meatus. At this position a soft tissue mass originating from the nasal mucosa was observed. There were no signs of an acute inflammation at this location. The mass filled out about a third of the lumen of the meatus and at least four living larvae were revealed in this tissue (Fig. 2). No larvae could be removed due to technical reasons. Examination of the left nostril was not carried out because bleeding and noticeable mucosal irritation occurred while examining the right nostril. Under general anaesthesia radiographs of the head were taken. No abnormal radiopaque structures could be found on the radiographs.

The alpaca was treated subcutaneously with doramectin by a dose of 0.2 mg/kg (Dectomax®, Lilly Deutschland GmbH, Elanco Animal Health, Bad Homburg, Germany).

**Fig. 3:**
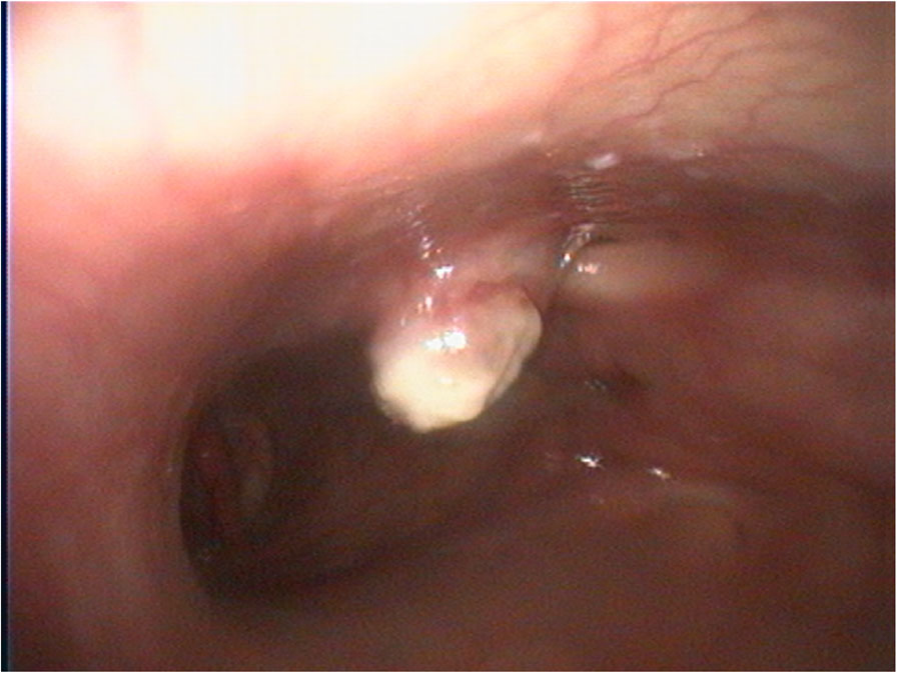
Endoscopic picture of the right ventral meatus from the affected alpaca after treatment. The soft tissue mass has decreased and no more larvae were found

Five days after treatment, sneezing could not be triggered any more by pressing on the bridge of the nose. Six days after treatment another endoscopic examination under local anaesthesia (Isocain ad us. vet.®) was carried out. Compared to the first examination, the soft tissue mass had notably decreased; there were no more larvae visible within the mass (Fig. 3).

In the endoscopy before the treatment with doramectin no larvae could be removed and, in addition, no larvae were sneezed out, so species of the bots could not be determined.

## Discussion

To the authors’ knowledge the present case is the first report of respiratory symptoms due to bot fly larvae in an alpaca. To date it is mentioned, that *Oestrus ovis* and *Cephenemyia* spp. are ectoparasites alpacas are exposed to [9], but no clinical symptoms are outlined. Additionally, it is the first published successful treatment of bot flies in New World camelids using doramectin.

In order to determine the causative species which has caused the alterations of the mucosa we tried to remove the larvae endoscopically. The larvae could not be gripped successful and additionally bleeding and swelling of the mucosa occurred. In some of the reported cases respiratory distress leading to a tracheotomy was caused by trying to remove larvae from the nostrils [2, 4]. To avoid such a serious intervention the attempt to remove the larvae was interrupted. Moreover, no larvae were found in the stable, so the species could not be identified. Both bot fly species which have been found in llamas (*Cephenemyia* spp. and *Oestrus ovis*) occur in Germany. The Camel nasal bot fly (*Cephalopina titillator*) occurs in large parts of Africa, Asia, Australia and the Middle East. Central Europe is not the natural habitat of this species [10]. An indication as to the identity of the causative bot fly species may be seen in the fact that the neutered male alpaca did not have any contact to sheep, but as it was kept on pasture with contact to deer, the end host of *Cephenemyia* spp.. Another hint to the causal species in the present case is the severe swelling within the nostril. It is known that *Cephenemyia* spp*.* in New World camelids cause a severe reaction, with noticeable swelling and granulation tissue [1, 4]. If it was *Oestrus ovis* the button in the posterior spiracular plate would be located in the center of the plate [10, 11]. In the photographs taken during endoscopy the buttons seem to be located at the medial border of the plate. This finding supports the suspicion, that the bot fly causing the myiasis could be *Cephenemyia* spp..

The small deviations from the reference range found in the haematology and clinical chemistry are not associated with the occurrence of bot fly larvae according to the current state of knowledge.

The formerly described cases of bot flies fly larvae in llamas were treated with ivermectin [2, 4], but this treatment was not successful after one administration in all cases. As alternative therapy we decided to use doramectin, as doramectin is an effective drug against myiasis due to bot flies in sheep. In sheep a dosage of 200 μg/kg has a sufficient effect on *Oestrus ovis* [6]. As such a dosage has no negative effects on alpacas [1] the alpaca in the present case was treated subcutaneously with doramectin 200 μg/kg. A single treatment was successful, the sneezing stopped, no larvae could be found within the nostril and the swelling decreased.

## Conclusion

This case report describes the first clinical finding of myiasis caused by bot flies in an alpaca. Additionally it is the first description of a successful treatment with doramectin. Bot fly larvae should be perceived as a differential diagnosis when an alpaca is presented with nonspecific respiratory symptoms, especially sneezing.
